# Synthetic Oxytocin and Vasopressin Act Within the Central Amygdala to Exacerbate Aggression in Female Wistar Rats

**DOI:** 10.3389/fnins.2022.906617

**Published:** 2022-05-18

**Authors:** Vinícius E. de M. Oliveira, Trynke R. de Jong, Inga D. Neumann

**Affiliations:** ^1^Laboratory of Neuroendocrinology, GIGA-Neurosciences, University of Liege, Liege, Belgium; ^2^Department of Neurobiology and Animal Physiology, Behavioural and Molecular Neurobiology, University of Regensburg, Regensburg, Germany; ^3^Medische Biobank Noord-Nederland B.V., Groningen, Netherlands

**Keywords:** female intruder test, aggression training, social isolation, amygdala, social behavior, aggression, vasopressin, oxytocin

## Abstract

Exacerbated aggression is a high-impact, but poorly understood core symptom of several psychiatric disorders, which can also affect women. Animal models have successfully been employed to unravel the neurobiology of aggression. However, despite increasing evidence for sex-specificity, little is known about aggression in females. Here, we studied the role of the oxytocin (OXT) and arginine vasopressin (AVP) systems within the central amygdala (CeA) on aggressive behavior displayed by virgin female Wistar rats using immunohistochemistry, receptor autoradiography, and neuropharmacology. Our data show that CeA GABAergic neurons are activated after an aggressive encounter in the female intruder test. Additionally, neuronal activity (pERK) negatively correlated with the display of aggression in low-aggressive group-housed females. Binding of OXT receptors, but not AVP-V1a receptors, was increased in the CeA of high-aggressive isolated and trained (IST) females. Finally, local infusion of either synthetic OXT or AVP enhanced aggression in IST females, whereas blockade of either of these receptors did not affect aggressive behavior. Altogether, our data support a moderate role of the CeA in female aggression. Regarding neuropeptide signaling, our findings suggest that synthetic, but not endogenous OXT and AVP modulate aggressive behavior in female Wistar rats.

## Introduction

Aggression is typically defined as a behavior that has the intention of inflicting physical harm to a conspecific (Nelson and Trainor, 2007; Masis-Calvo et al., 2018; Lischinsky and Lin, 2020). In nature, aggression is adaptive and expressed whenever animals engage in conflict to get access to essential resources for their survival, such as territory, food, or mates (Nelson and Trainor, 2007; Hashikawa et al., 2018; Masis-Calvo et al., 2018). However, aggression may also be expressed out of context or excessively. In humans, such abnormal aggressive behavior is listed as a core symptom of several psychiatric disorders, such as conduct disorder, anti-social personality disorder, schizophrenia, bipolar disorder, and post-traumatic stress disorder (Nelson and Chiavegatto, 2001; Glenn et al., 2013; Reidy et al., 2015; Haller, 2016). The fact that aggression often becomes pathological (Glenn et al., 2013; Reidy et al., 2013, 2015), associated with its potential to cause a burden to society (Sourander et al., 2007; Reidy et al., 2015; Sumner et al., 2015), has driven scientists to use model organisms such as songbirds, rodents, cats and primates to study the neurobiology of aggressive behavior (Lischinsky and Lin, 2020). Interestingly, most of these models, and therefore studies, were conducted on males (De Almeida et al., 2005; Koolhaas et al., 2013; Haller, 2016). In contrast, abnormal aggression in virgin females – aside from the adaptive and highly context-specific behavior known as maternal aggression (Bosch, 2013; Hashikawa et al., 2018; Lischinsky and Lin, 2020) – has only recently gained more attention (Smaragdi et al., 2017; Freitag et al., 2018; Ackermann et al., 2019).

The lack of scientific interest in female aggression is largely derived from the misconception that females in most species, including humans, are rarely or very mildly aggressive. Recent studies, however, have shown that girls and women exihibit disruptive aggression and can develop aggression disorders similarly to boys and men (Campbell, 1999; Mancke et al., 2015; Denson et al., 2018; Freitag et al., 2018; Ackermann et al., 2019), thereby negatively impacting their own quality of life and of their victims. Furthermore, studies in rodents indicated that females are able to show a considerable amount of aggression (Silva et al., 2010; Staffend and Meisel, 2012; Been et al., 2016, 2019; Hashikawa et al., 2017; Newman et al., 2019; Oliveira et al., 2019, 2021) and that the neurobiological mechanisms regulating aggressive behavior might be sex-dimorphic (Ho et al., 2001; Silva et al., 2010; Cordero and Sandi, 2013; Terranova et al., 2016; Hashikawa et al., 2017, 2018; Borland et al., 2019; Newman et al., 2019; Oliveira et al., 2019, 2021). Altogether, this highlights the need to learn more about the neurobiology of female aggression in order to identify possible targets of treatment for women and girls.

Here, we used our established model of exacerbated aggression in female Wistar rats (Oliveira et al., 2021) to assess the role of the oxytocin (OXT) and arginine vasopressin (AVP) neuromodulation within the central amygdala (CeA) in aggression. The amygdalar complex has been substantially linked with aggression in male rodents (Haller, 2013, 2018) and humans (Rosell and Siever, 2015). Specifically, morphological (Matthies et al., 2012) as well as functional (da Cunha-Bang et al., 2017) changes in the amygdala have been associated with high aggression in male offenders and boys with conduct disorder, respectively. In rodents, a participation of the amygdalar complex on aggression is also likely (Haller, 2018). More precisely, CeA hyperactivation was found after aggressive encounters in male rats (Toth et al., 2012) and in rat models of exaggerated and abnormal aggression such as post-weaning social isolation (Toth et al., 2012) and peripubertal stress (Marquez et al., 2013).

The OXT and AVP systems have also been extensively implicated in the regulation of aggression in rodents in both sexes, often showing antagonistic effects (Landgraf and Neumann, 2004; Elliott Albers et al., 2006; Veenema et al., 2010; Bosch and Neumann, 2012; De Jong et al., 2014; Terranova et al., 2016; Lukas and de Jong, 2017; Oliveira et al., 2021). Especially, the behavioral effects of OXT on aggression are suggested to be partly mediated *via* the CeA. Extremely aggressive male wild-type Groningen rats exhibited increased OXT receptor (OXTR) binding compared to low aggressive controls in the CeA, and OXTR binding positively correlated with aggressive behavior (Calcagnoli et al., 2014). Conversely, intra-CeA infusion of synthetic OXT in those highly aggressive rats decreased aggression (Calcagnoli et al., 2015). In lactating females, the opposite has been described as both peptides were released during maternal aggression (Bosch et al., 2005; Bosch and Neumann, 2010). Additionally, lactating females, which are known to display high levels of maternal aggression, presented elevated OXTR (Bosch et al., 2005) as well as vasopressin 1a (V1aR) receptor (Caughey et al., 2011) binding in the CeA.

Based on this evidence we hypothesized that the CeA, and more specifically local OXT and AVP neurotransmission acting *via* OXTR and V1aR, respectively, would play a role in the aggressive behavior displayed by virgin female rats. To test this hypothesis we used low aggressive group-housed (GH) and highly aggressive isolated and trained (IST) virgin female rats as model organisms (Oliveira et al., 2021). First, we evaluated, whether CeA GABAergic neurons are recruited by the display of high levels of aggression in IST females. Next, we investigated, whether GH and IST females showed differences in local OXTR and V1aR binding. Finally, we assessed the effects of activation vs. blockade of OXTRs and V1aRs within the CeA on various aspects of aggressive behavior in the female intruder test (FIT), using neuropharmacology.

## Materials and Methods

### Animals

Experimental animals were adult virgin female Wistar and Venus-VGAT [VGAT: vesicular GABA transporter; lineVenus-B, W-Tg(Slc32a1-YFP*)1Yyan, Wistar background] (Uematsu et al., 2008) rats (10–14 weeks old) bred in the animal facilities of the University of Regensburg (Germany). Stimulus (intruder) animals were adult female Wistar rats obtained from Charles Rivers Laboratories (Sulzfeld, Germany) kept in groups of 3 to 5 animals in a separate animal room. All subjects were kept under controlled laboratory conditions (12:12 h light/dark cycle; lights off at 11:00, 21 ± 1°C, 60 ± 5% humidity) with access to standard rat nutrition (RM/H, Sniff Spezialdiäten GmbH, Soest, Germany) and water *ad libitum*. All behavioral procedures were conducted following the Guidelines for the Care and Use of Laboratory Animals of the Local Government of Oberpfalz and Unterfranken.

### Housing Conditions

As previously described (Oliveira et al., 2021) experimental females were kept in two different housing conditions: GH females were kept in groups of 3 to 5 animals per cage, whereas IST females were kept socially isolated for 8 days except for the time of exposure to the FIT, performed on 3 consecutive days according to the aggression training protocol starting on day 5 after social isolation. Approximately 4 h prior the FIT GH females were also placed in observation cages for behavioral testing.

### Female Intruder Test

The FIT was performed as previously described (De Jong et al., 2014; Oliveira et al., 2021). Briefly, an unfamiliar female intruder weighing between 10 and 20% less than the female resident was placed into the resident’s home cage for 10 min in the early dark phase (between 12:00 and 17:00) under dim red light conditions. All the resident’s behaviors during the FIT were analyzed live (pen and paper scoring) and videotaped for later detailed analysis by an experienced observer blind to treatment, using JWatcher event recorder Program (Blumstein et al., 2000). The observed behaviors were pooled in four behavioral domains: (i) aggressive behaviors (i.e., keep down, threat, offensive grooming, and attacks); (ii) neutral behaviors (i.e., exploring the home cage, drinking, eating, and self-grooming); (iii) social behaviors (i.e., non-aggressive social interactions, sniffing, following); and (iv) defensive behaviors (i.e., escaping from the intruder, defensive posture, and kicking the intruder with the hindlimb), and quantified as the percentage of time that the resident displayed the four types of behavior. The resident’s but not intruder’s estrous cycle was monitored *via* daily vaginal smears, taken directly after the FIT. All phases of the estrous cycle were included in the analysis, but no significant effect was found, as previously shown (Oliveira et al., 2021).

### Experimental Design

For detailed animal numbers per experiment please see Table 1.

**TABLE 1:** Overview of detailed statistical analysis as well as *p* values for all data displayed in Figures 1–4.

Figures	Data	Detailed statistics	*p* value	Numbers
1	Aggression (A)	two-tailed Student’s *t*-test *t*_(18)_ = 3.76	**0.001**	*N* = 10
	VGAT (B)	two-tailed Student’s *t*-test *t*_(18)_ = 0.23	0.822	
	pERK (B)	two-tailed Student’s *t*-test *t*_(18)_ = 1.33	0.200	
	VGAT/pERK (*n*; B)	two-tailed Student’s *t*-test *t*_(18)_ = 2.087	0.051	
	VGAT/pERK (%; C)	two-tailed Student’s *t*-test *t*_(18)_ = 2.102	**0.049**	
2	Aggression (A)	two-tailed Student’s *t*-test *t*_(14)_ = 5.410	**<0,0001**	GH = 10; IST = 7
	OXTR binding (B)	two-tailed Student’s *t*-test *t*_(13)_ = 2.47	**0.028**	GH = 8; IST = 7
	V1aR binding (C)	two-tailed Student’s *t*-test *t*_(14)_ = 0.0003	0.999	GH = 10; IST = 6
3	Total aggression (A)	one-way ANOVA followed by Bonferroni *F*_(3,36)_ = 7.27	**0.006**	VEH = 12; OXT = 8; TGOT = 7; AVP = 13
	Keep down (A)	Kruskal–Wallis test followed by Dunn’s *H*_4_ = 14.08	**0.002**	
	Threat (A)	Kruskal–Wallis test followed by Dunn’s *H*_4_ = 2.21	0.592	
	Off. Grooming (A)	Kruskal–Wallis test followed by Dunn’s *H*_4_ = 1.44	0.697	
	Latency to attack (C)	Kruskal–Wallis test followed by Dunn’s *H*_4_ = 14.09	**0.003**	
	Number of attacks (D)	Kruskal–Wallis test followed by Dunn’s *H*_4_ = 14.08	**0.003**	
	Exploring (B)	one-way ANOVA followed by Bonferroni *F*_(3,36)_ = 2.62	0.065	
	Social (B)	one-way ANOVA followed by Bonferroni *F*_(3,36)_ = 2.84	**0.05**	
	Grooming (B)	one-way ANOVA followed by Bonferroni *F*_(3,36)_ = 0.38	0.628	
	Drinking and eating (B)	Kruskal–Wallis test followed by Dunn’s *H*_4_ = 10.59	**0.014**	
4	Total aggression (A)	one-way ANOVA followed by Bonferroni *F*_(2,30)_ = 0.68	0.512	VEH = 11; OXTR-A = 11; V1aR-A = 11
	Keep down (A)	Kruskal–Wallis test followed by Dunn’s *H*_3_ = 0.81	0.667	
	Threat (A)	Kruskal–Wallis test followed by Dunn’s *H*_3_ = 0.25	0.883	
	Off. Grooming (A)	Kruskal–Wallis test followed by Dunn’s *H*_3_ = 0.6	0.740	
	Latency to attack (C)	Kruskal–Wallis test followed by Dunn’s *H*_3_ = 0.81	0.667	
	Number of attacks (D)	Kruskal–Wallis test followed by Dunn’s *H*_3_ = 0.45	0.799	
	Exploring (B)	one-way ANOVA followed by Bonferroni *F*_(2,30)_ = 0.35	0.706	
	Social (B)	one-way ANOVA followed by Bonferroni *F*_(2,30)_ = 0.14	0.868	
	Grooming (B)	Kruskal–Wallis test followed by Dunn’s *H*_3_ = 0.91	0.633	
	Drinking and eating (B)	Kruskal–Wallis test followed by Dunn’s *H*_3_ = 10.67	0.716	

*Significant p values in bold. AVP, vasopressin; GH, group-housed; IST, Isolated and trained; OXT, oxytocin; OXTR-A, OXT receptor antagonist; TGOT, selective OXT receptor agonist; VEH, vehicle; VGAT, vesicular GABA transporter; and V1aR-A, V1a receptor antagonist.*

**FIGURE 1:**
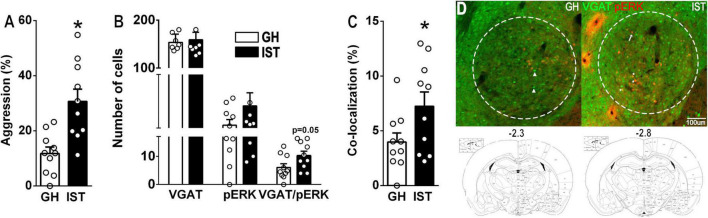
Social isolation and aggression-training (IST, black bars) exacerbate the percentage of time group-housed (GH, white bars) female Venus-VGAT rats spent on aggression (sum of time spent on keep down, threat, offensive grooming, and attacking/total time × 100%) in a 10-min long FIT **(A)**. In IST females we found an increased number of cells showing co-localization of VGAT (green) and pERK (red) in the central amygdala **(B–D)**. **(D)** Maximal *z*-projection of VGAT (green) and pERK (red). Scale bar 100 μm. Data are presented as mean + s.e.m. *p < 0.05 vs. GH. Arrow-heads, arrows, and stars to exemplify VGAT-, pERK-, and VGAT-pERK-positive cells, respectively.

In experiment 1 (*neuronal activity*, Figure 1), we aimed to evaluate, whether GABAergic neurons within the CeA are differentially recruited by the display of low vs. high aggression in female rats. Therefore, Venus-VGAT females were kept in either GH or IST housing conditions for 8 days (section “Housing Conditions”). On day 9, 1 day after the last training session of the IST animals, rats of both groups were exposed to the FIT in their home cage (FIT 4, for IST females and FIT1 for GH females, section “Housing Conditions”). Directly after being exposed to the FIT, GH and IST rats were transcardially perfused and brains were processed for pERK immunohistochemistry (section “Immunohistochemistry”).

**FIGURE 2:**
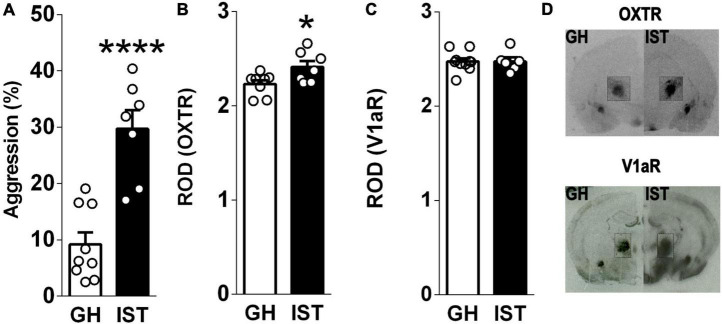
Isolated and trained (IST) female Wistar rats showed exacerbated aggression **(A)** accompanied by increased oxytocin (OXTR, **B, D**, insert) but not vasopressin 1a (V1aR, **C, D**, insert) receptor binding in the central amygdala. Data are shown as relative optical density (ROD). Data presented as mean + s.e.m. *p < 0.05, ****p < 0.0001 vs GH.

In experiment 2 (*receptor binding*, Figure 2), we aimed to verify, whether distinct aggression phenotypes are associated with differential OXTR and V1aR binding in the CeA of virgin female Wistar rats. Therefore, rats were exposed to either GH or IST housing conditions (section “Housing Conditions”). One day after the last training of IST females (day 9), both GH and IST females received a FIT to confirm their levels of aggression. Immediately after the FIT, animals were processed for receptor autoradiography (section “Receptor Autoradiography”).

For experiments 3 (*agonists)* and 4 (*antagonists*), only IST females were used. After recovery from stereotaxic surgery (section “Stereotaxic Surgery,” day 5) rats underwent the aggression training protocol (section “Housing Conditions”) accompanied by a sham-infusion (i.e., having a 12 mm infusion system placed into the guide cannulae without having fluid injected into the brain, please note that the infusion system did not reach/lesion the CeA as they had the same size of the guide cannulae section “Stereotaxic Surgery”) before each FIT to adapt to the infusion procedure. In order to guarantee similar levels of aggression among the treatments, the average level of aggression during the 3rd FIT (last day of training protocol) was used to equally allocate IST rats into the treatment groups (not shown). As we found increased OXTR binding in highly aggressive IST in experiment 2 (Figure 2), we tested the effects of *synthetic* OXT on aggression displayed by highly aggressive IST females. Additionally, we assessed the effects of synthetic AVP infusion into the CeA to control for receptor cross-reactivity. Finally, for experiment 4 (Figure 4), we evaluated, whether pharmacological blockade of OXTR and V1aR affects aggression in virgin female rats.

### Stereotaxic Surgery

Stereotaxic surgery was performed under semi-sterile conditions as previously described (De Jong et al., 2014; Oliveira et al., 2021). Briefly, rats were anesthetized with isoflurane, injected i.p. with the analgesic Buprenovet (0.05 mg/kg Buprenorphine, Bayer, Germany) and the antibiotic Baytril (10 mg/kg Enrofloxacin, Baytril, Bayer, Germany), and mounted in the stereotaxic frame. Intra-CeA (AP: -2.5; ML: ±4.0; DV: +6.0) guide cannulas (25 G, 12 mm; Injecta GmbH, Germany) were implanted bilaterally, 2 mm above the target region to avoid lesioning. Local cannulas were fixed to the skull with two jeweler’s screws and dental cement (Kallocryl, Speiko-Dr. Speier GmbH, Muenster, Germany) and closed by a stainless steel stylet (27 G). Cannulated females were left single-housed and undisturbed for 5 days to recover, prior to the start of the aggression training protocol.

### Immunohistochemistry

Experimental animals were deeply anesthetized with isoflurane followed by CO_2_ and transcardially perfused with paraformaldehyde (PFA) 4%. Following perfusion, brains were removed, kept in PFA 4% overnight (postfixation), dehydrated in sucrose 30%, and snap-frozen before cryocutting. Slices (40 μm) containing the CeA (Bregma −2.3 to −2.8), were collected in cryoprotectant solution and stored at −20°C until the experiment took place. A series of 6–8 slices were used for immunostaining. First, slices were washed in 0.1 PBS, incubate in ice-cold methanol at −20°C for 10 min, then rinsed in Glycine buffer (0.1M in PBS) for 20 min. Afterward, slices were washed with PBST (0.1 PBS with 0.3% triton-× 100) and blocked for 1 h in a solution of normal goat serum (NGS 5%). Directly after blocking, slices were incubated in primary antibodies rabbit-anti-pERK antibody (1:250 CellSignalling #4370, Danvers, MA, United States) at 4°C for 64 h. Next, slices were left at room temperature for 1–2 h, washed in PBST, and incubated with the secondary antibody Alexa-fluor 594 goat-anti-rabbit antibody (1:200, ThermoFisher Scientific, Germany). After secondary antibody incubation, brain sections were rinsed in 0.1 PBS and mounted on adhesive microscope slides (Superfrost Plus, Thermo Fisher Scientific Inc, United States). Slides were kept in the dark at 4°C until imaging. Imaging was done using an inverted confocal laser scanning microscope (Leica TCS SP8, Leica Microsystems, Wetzlar, Germany). Digital images were processed (Merging and *Z*-projections) using the Leica Application Suite × (Leica) and Fiji70. Cell counting was done by an experienced observer blind to the treatments (Schindelin et al., 2012). All positive neurons for pERK and VGAT within the CeA region were counted. Importantly, area delimitation used for quantification did not differ between the groups.

### Receptor Autoradiography

Experimental animals were euthanized *via* intraperitoneal injection of urethane (25%, 1.2 ml/kg), followed by decapitation. Brains were removed and snap-frozen for cryocutting and receptor autoradiography analysis. Coronal brains sections (Bregma −2.3 to −2.8, 16-μm) were slide-mounted and stored at −20°C. The receptor autoradiography procedure was performed using a linear V1a-R antagonist [125I]-d(CH2)5(Tyr[Me])-AVP (Perkin Elmer, United States) or a linear OXTR antagonist [125I]-d(CH2)5[Tyr(Me)2-Tyr-Nh2]9-OVT (Perkin Elmer, United States) as tracers, as described (Oliveira et al., 2019, 2021). Slides were thawed and dried at room temperature followed by a short fixation in paraformaldehyde (0.1%). Next, samples were washed two times in 50 mM Tris (pH 7.4), exposed to tracer buffer (50 pM tracer, 50 mM Tris, 10 mM MgCl2, 0.01% BSA) for 60 min, washed four times in Tris buffer 10 mM MgCl2, shortly dipped in pure water, and dried at room temperature overnight. On the next day, slides were exposed to Biomax MR films for 20 days (Kodak, Cedex, France). Then, films were scanned using an EPSON Perfection V800 Scanner (Epson, Germany). FijiImageJ (V1.37i, National Institute of Health, http://rsb.info.nih.gov/ij/) was used for optical analysis (Schindelin et al., 2012). Receptor density was calculated by sampling the target region, average gray density was calculated after subtracting the tissue background. Gray density was then converted to relative optical density as previously described (Baskin and Stahl, 1993). Bilateral measurements of 6 brain sections were analyzed for each rat.

### Drugs

For neuropharmacology experiments, rats were either treated with the nonapeptide agonists [OXT: 10 ng, AVP: 0.1 ng, or TGOT, (Thr4,Gly7)OT: 10 ng/0.5 μl per side, Tocris] (Huber et al., 2005; Manning et al., 2012; Busnelli et al., 2013; Oliveira et al., 2021) 5 min prior to the FIT or with the selective receptor antagonists (OXTR-A and V1aR-A: 100 ng/0.5 μl, per side, Tocris; Oliveira et al., 2021) 10 min before the FIT. Cannula placement was confirmed *via* post mortem injection of blue ink followed by Nissel staining, hence infusion sites outside of the CeA amygdala were not included in the statistical analysis.

### Statistics

Data normality was assessed using the Kolmogorov–Smirnov test. If normality was reached, data were analyzed using either Student’s *t*-test (unpaired) or analyses of variance (one-way ANOVA) followed by a *post hoc* comparison corrected with Bonferroni, when appropriate. Otherwise, either Mann–Whitney *U*-tests or Dunn’s multiple comparison tests were performed. Pearson’s and Spearman’s correlations were used to correlate pERK and pERK/VGAT cell countings with behavioral data. For detailed statistical information see Table 1.

## Results

### Highly Aggressive Females Show a Moderate Increase in Activity of GABAergic Neurons in the Central Amygdala

Social isolation and aggression training robustly increased the percentage of the time female Wistar rats engaged in aggressive behavior during the FIT (Figure 1A). Specifically, IST rats spend more time on keep down, threat, and attack compared to GH females, as well as showed a higher number of attacks and a shorter attack latency (Table 2) thus confirming recent results and demonstrating the robustness of the IST protocol to elevate aggression in female rats (Oliveira et al., 2021).

**TABLE 2:** Detailed analysis of aggressive behaviors displayed during the FIT by isolated and trained (IST) and group-housed (GH) females (Experiment 1 and Figure 1) *n* = 10.

Behavior	GH (mean ± SEM)	IST (mean ± SEM)	*p* value	Statistics
Keep down (%)	1.573 ± 0.738	17.04 ± 4.120	**0.0014**	Two-tailed student’s *t*-test
Threat (%)	2.448 ± 0.79	9.017 ± 1.297	**0.0004**	Two-tailed student’s *t*-test
Offensive grooming (%)	2.826 ± 0.584	8.192 ± 1.746	**0.0093**	Two-tailed student’s *t*-test
Attack time (%)	0.098 ± 0.087	0.8182 ± 0.2673	**0.0017**	Mann–Whitney *U*-tests
Attack number (*n*)	0.3 ± 0.213	4.7 ± 1.044	**0.0004**	Mann–Whitney *U*-tests
Latency to attack (s)	557.8 ± 28.17	267.8 ± 57.45	**0.0005**	Mann–Whitney *U*-tests

*Significant p values in bold.*

IST compared with GH females tended to exhibit a higher number of VGAT/pERK-positive cells after FIT exposure (Figure 1B). Consequently, the percentage of co-localization (VGAT-positive cells expressing pERK/total number of VGAT cells × 100) was elevated in IST compared to GH rats (Figures 1C,D). However, no consistent correlation was found between pERK^+^ or pERK/VGAT^+^ cell numbers, or percentage of colocalization and total time spend on aggression (%), when all animals were pooled. Notably, within the GH group aggression negatively correlated with the absolute number of pERK^+^ cells. Interestingly, the same trend was observed in IST females, when aggression was correlated with the percentage of colocalization (pERK^+^VGAT^+^/VGAT^+^ × 100%; Table 3).

**TABLE 3:** Overview of correlation coefficients between aggressive behavior, displayed by group-housed (GH) or isolated and trained (IST) females during the FIT, and the number of pERK^+^ cells and pERK^+^/VGAT^+^ (number of cells and percentage of colocalization).

Group	Parameters					
	pERK (*n*)		pERK/VGAT (*n*)		pERK/VGAT (%)	
	*r*	*p* value	*r*	*p* value	*r*	*p* value
GH	−0,6375	**0,0474**	−0,002357	0,9948	0,07300	0,8412
IST	−0,5641	0,0894	−0,01343	0,9706	−0,6165	0,0577*
Combination	−0,2316	0,3259	−0,3519	0,1281	−0,2704	0,3259

*GH and IST data were analyzed via Pearson’s correlation. The combination of the two housing conditions was analyzed via Spearman’s correlation. Significant correlations in bold.*

*Nearly significant.

### Oxytocin, but Not V1a Receptor Binding Is Increased in Highly Aggressive Isolated and Trained Females

Highly aggressive IST females (Figure 2A) showed elevated OXTR binding in the CeA compared to GH controls (Figures 2B,D). In contrast, V1aR binding in the CeA did not differ between IST and GH rats (Figures 2C,D).

### Synthetic Oxytocin and Arginine Vasopressin Infusion Into the Central Amygdala Exacerbate Female Aggression in Rats

**FIGURE 3:**
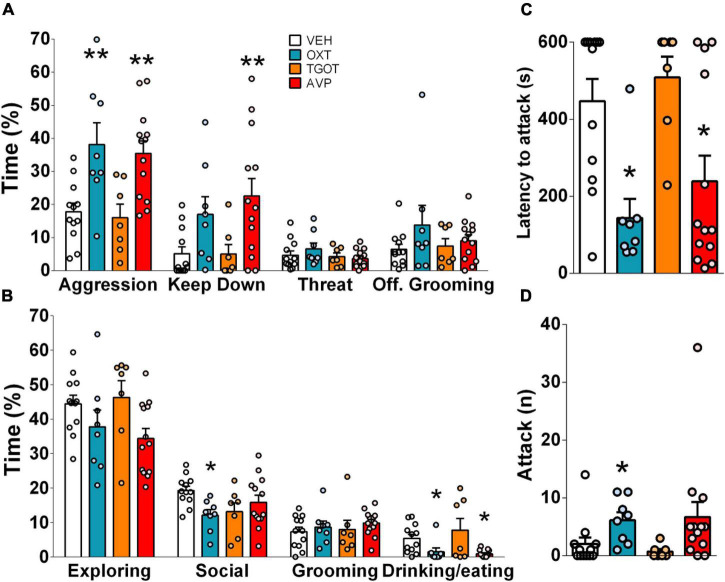
Infusion of synthetic vasopressin (AVP, red bars, 0.1 ng/0.5 μl per side) and oxytocin (OXT, blue bars, 10 ng/0.5 μl per side) but not TGOT (selective OXTR agonist, orange bars, 10 ng/0.5 μl, per side), into the central amygdala increased percentage of time spent on total aggression in IST rats **(A)**. Particularly, AVP increased the percentage of time spent on total aggression and keep down **(A)**, and reduced the latency to attack **(C)**. Whereas OXT heightened the percentage of time spent on total aggression **(A)**, the number of attacks **(D)**, and decreased the latency to attack **(C)** and the percentage of time spent on the non-aggressive social investigation **(B)**. Both peptides reduced the percentage of time spent feeding and drinking. Data presented as mean + s.e.m. *p < 0.05 vs. VEH; **p < 0.01 vs. VEH.

Infusion of either synthetic OXT or AVP, but not of TGOT (a selective OXTR agonist), significantly enhanced the high levels of aggression seen in IST rats (Figure 3A). Interestingly, although both peptides significantly decreased the latency to attack (Figure 3C), only OXT elevated the number of attacks (Figure 3D), whereas only AVP increased time spent on keep down (Figure 3A). Surprisingly, synthetic OXT reduced the display of non-aggressive social behaviors in IST females (Figure 3B). In addition, both synthetic OXT and AVP mildly decreased the percentage of time animals spent feeding and drinking in their home cage (Figure 3B). Neutral behaviors such as grooming and exploratory behaviors were not affected by the treatments. Please note that no consistent behavioral effect was found when infusion sites were located outside of the CeA.

**FIGURE 4:**
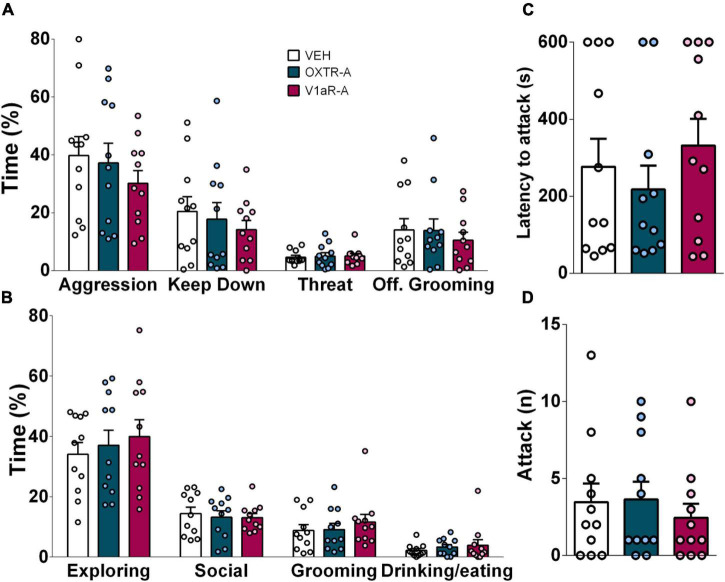
Infusion of either OXT receptor antagonist (OXTR-A, dark blue bars) or V1a receptor antagonist (V1aR-A, dark pink, both at 100 ng/0.5 μl per side) into the CeA did not affect **(A)** aggressive behavior, neutral or social behaviors **(B)**, or **(C)** attack latency, **(D)** the number of attacks displayed by virgin female IST rats. Data presented as mean + s.e.m.

### Blockade of Oxytocin and V1a Receptors in the Central Amygdala Does Not Affect Female Aggression

Neither blockade of OXTR nor V1aR in the CeA prior to the FIT affected the display of aggression by female IST rats. Furthermore, social, neutral, and defensive behaviors remained unchanged after the treatment (Figure 4).

## Discussion

The present study demonstrates that the CeA plays a moderate role in the regulation of aggressive behavior displayed by female Wistar rats. Specifically, our findings reveal that the display of high levels of aggression, during the FIT, elevated the activity of GABAergic neurons in the CeA, as IST females exhibited a higher percentage of colocalization (pERK/VGAT^+^/VGAT^+^ × 100) compared to GH controls. Interestingly, a negative correlation was found between pERK-positive cells and aggression in GH rats indicating that in low aggressive animals activation neurons in the CeA are rather related to an inhibitory effect on female aggression. A similar trend was observed in IST females as aggression tended to negatively correlate with the percentage of colocalization (pERK^+^VGAT^+^/VGAT^+^ × 100%). Moreover, we could reveal the role of the OXT and AVP systems within the CeA in female aggression. In detail, OXTR, but not V1aR binding was found to be elevated as a consequence of social isolation and aggression training which we hypothesize to be associated with the high levels of aggression observed in IST rats. Accordingly, infusions of synthetic OXT and AVP into the CeA exacerbated the (already high) levels of aggression of IST females. Particularly synthetic OXT reduced the display of social behaviors, such as social investigation and approach. Surprisingly, blockade of OXTR or V1A receptors did not alter the performance of IST female rats in the FIT, implying that endogenous release is not necessary for the display of aggression in female rats.

The participation of the amygdalar complex in aggression is controversial. Studies in humans have linked structural as well as activity changes in this area with disruptive violent behavior in males (Rosell and Siever, 2015). Typically atrophy of the amygdala is associated with high levels of aggression in healthy male participants (Matthies et al., 2012). However, contrasting evidence has been found in functional studies, as violent offenders exhibited hyperactivation of the amygdala after provocation (da Cunha-Bang et al., 2017). Unfortunately, data on the participation of the amygdala in disruptive aggressive behavior displayed either by violent or healthy women are still missing in the literature, thereby a parallel with our findings can not be easily drawn.

Concerning rodent models of aggression, a similar ambiguous picture, mainly arising from studies performed in male or female lactating rodents, is found. Classically, the CeA is known to be associated with predatory aggression rather than intermale or “rivalry”-related aggression (Haller, 2013, 2018; Han et al., 2017). Nevertheless, the display of aggression itself during the resident intruder test seems to activate the CeA in male Wistar rats (Toth et al., 2012). Furthermore, high activity of the CeA has been reported after a resident intruder-test in male rat models of abnormal aggression such as post-weaning social isolation (Toth et al., 2012), rats bred for low-anxiety related behavior (LAB; Veenema et al., 2007; Beiderbeck et al., 2012), and peripubertal stress (Marquez et al., 2013). Additionally, highly aggressive lactating mice also show increased c-Fos and Egr-1 in the CeA after the display of maternal aggression (Hasen and Gammie, 2005, 2006). Haller (2018) hypothesized that low arousal states such as the ones seen in male LAB rats (Veenema et al., 2007) or after peripubertal stress (Marquez et al., 2013) lead to a hyperactivation of the CeA. In fact, our data partially support this hypothesis as female aggression was found to negatively correlate with plasma corticosterone levels, which tended to be lower in highly aggressive IST rats (Oliveira et al., 2021). Altogether, our neuronal activity findings add an important piece of evidence to this hypothesis by demonstrating that the CeA seems to be recruited by exposure to an intruder in the FIT, although to a moderate extent.

Nevertheless, the mismatch between absolute neuronal activity differences (% of colocalization, higher in IST females) and the negative correlations found in both groups remains elusive. The amygdala complex (especially the basolateral amygdala → CeA pathway) has been implicated in processes such as valence encoding (Janak and Tye, 2015) and empathy/emotion recognition (Klein and Gogolla, 2021). In fact, OXT neurons projecting from the paraventricular nucleus of the hypothalamus to the CeA as well as OXTR expression locally seem to be essential for social discrimination of emotional states in male and female mice (Ferretti et al., 2019). Thus, we hypothesize that the absolute differences in neuronal activity within the CeA with increased pERK activity in IST females might rather reflect the response of the resident’s CeA (presumably OXTR-expressing neurons) to the negative cues (distress, dominance rank, and social status) of the intruder during the FIT. Also, social isolation itself may exacerbate the perception of these cues in IST females, as activated neurons might be more sensitive to social interactions. Future studies should investigate, whether the CeA (especially OXTR-expressing neurons) of highly aggressive IST females responds differentially to distinct intruder cues and whether these responses are associated with their levels of aggression and/or social isolation.

Regarding neuropeptide signaling, our finding of elevated OXTR, but not V1aR binding in highly aggressive IST rats is supported by two studies, one performed in male Groningen rats displaying exaggerated aggression (Calcagnoli et al., 2014) and the other performed lactating rats where OXTR binding was increased in highly aggressive dams (Bosch et al., 2005). In excessively aggressive male rats, OXTR binding positively correlated with aggression, indicating that increased OXT signaling stimulates aggression (Calcagnoli et al., 2014). Additionally, OXTR binding in the CeA amygdala was negatively correlated with social investigation in female Wistar rats (Dumais et al., 2013), reinforcing a potential pro-aggressive and/or antisocial role of OXT within the CeA in females.

Importantly, our data reveal a sex-specific role of synthetic OXT within the CeA in the context of aggression. Specifically, we have shown that infusion of synthetic OXT into the CeA further heightens aggression and reduces social approach in virgin female Wistar rats, whereas in male wild-type Groningen rats intra-CeA infusion of OXT was found to decrease aggression and increase time spend on social behavior (Calcagnoli et al., 2015). Furthermore, we have also observed a similar effect of enhanced aggressive behavior after local infusion of synthetic AVP. This strongly indicates cross-reactivity of AVP binding OXTR (higher binding in IST females), or OXT to V1aRs. Based on our results, the latter hypothesis is more likely, as the selective OXTR agonist TGOT did not increase female aggression after infusion into the CeA. In fact, we and others have already reported binding of OXT to V1aRs in the context of aggression and social behaviors (Song et al., 2014; Tan et al., 2019; Oliveira et al., 2021). However, it is important to point out that synthetic OXT and AVP affected different subtypes of aggressive behaviors displayed during the FIT. For instance, only OXT affected the *number of attacks*, whereas *keep down* was exclusively affected by AVP. Furthermore, only OXT influenced the percentage of time spent on *social investigation*. The lack of effect of TGOT on female aggression may also be due to the lower affinity of TGOT to OXTRs compared to synthetic OXT (Manning et al., 2012; Busnelli et al., 2013; Tan et al., 2019), thereby future studies should assess the effect of higher doses of TGOT on female aggression or combine administration of OXT with OXTR-A.

Concerning the pro-aggressive effect of synthetic AVP in female aggression, it needs to be mentioned that from the best of our knowledge our study is the first assessing the effect of intra-CeA infusion of synthetic AVP on territorial/”rivalry”-related aggression. A similar pro-aggressive effect was observed in lactating LAB rats, as an infusion of synthetic AVP enhanced maternal aggression during the maternal defense test (Bosch and Neumann, 2010). Therefore, our study highlights the participation of synthetic AVP in female aggression regardless of the reproductive state of the female.

Finally, blockade of both receptors *via* selective antagonists prior to the FIT did not affect aggression displayed by IST females. This is in agreement with male data, as OXTR-A infusion into the CeA was found to not affect aggressive or social behaviors in wild-type Groningen rats (Calcagnoli et al., 2015), suggesting that local peptide release is not required during the display of aggressive behavior in both sexes. However, in lactating HAB rats blockade of both receptors (OXTR and V1aR) was found to reduce maternal aggression (Bosch et al., 2005; Bosch and Neumann, 2010), indicating that endogenous peptide release during aggression might be reproductive state-dependent. Thus, in contrast with synthetic, endogenous peptides do not seem to be necessary for the display of territorial aggression in both sexes. Surprisingly, the same does not apply to maternal aggression, indicating that the involvement of peptides in female aggression might change due to alterations in the reproductive state.

Here it should be acknowledged that the high volume (0.5 μl) infused into the CeA may have difused into adjacent nuclei, thus participation of other amygdalar nuclei is possible although unlikely. Especially considering that only infusion sites located within the CeA region were included in the analysis, and no consistent behavioral effects of any of the treatments were found in the misses (outside of the CeA). Finally, similar volumes have been shown to act specifically before (Bosch et al., 2005; Bosch and Neumann, 2010; Lukas et al., 2011; Knobloch et al., 2012).

We have also found that both synthetic OXT and AVP decreased the time spent feeding and drinking in female Wistar rats. Indeed, neuronal populations within the CeA have been implicated in the control of predatory (Han et al., 2017) and normal (Douglass et al., 2017) feeding behaviors. Additionally, stimulation of OXT neurons in the paraventricular nucleus of the hypothalamus, as well as intracerebroventricular infusion of synthetic AVP, are known to suppress feeding in male mice and rats, respectively (Atasoy et al., 2012; Santoso et al., 2018). Here we have shown that similar effects are seen in females, as both peptides reduced the percentage of time spent feeding and drinking during an encounter with a con-specific (FIT). Perhaps the same population of OXT and AVP-responsive neurons in the CeA are controlling different appetitive and consummatory behaviors such as fighting and feeding. Future studies should dissect, whether OXT and AVP signaling within CeA also decrease food and water consumption in specific behavioral tests and whether the same neuronal populations control both types of behavior.

## Conclusion

Altogether our data points toward rather moderate participation of the CeA in the regulation of aggression in female Wistar rats. Interestingly, in contrast to our previous results in the lateral septum showing antagonistic effects of OXT and AVP (Oliveira et al., 2021), here we have demonstrated that both neuropeptides seem to act synergistically within the CeA to enhance aggression.

Additionally, we have confirmed that administration of synthetic AVP into the CeA enhances aggression also in virgin females supporting our previous results in lactating dams (Bosch and Neumann, 2008). Regarding OXT, our data adds an important piece of evidence to the sex-specific effects of OXT in the context of aggression and highlights the importance of conducting studies in both sexes, as we have confirmed that local infusion of synthetic OXT differentially affects aggression and social investigation in male (Calcagnoli et al., 2015) and female (this study) rats.

## Data Availability Statement

Data sets will be made available upon reasonable request to the corresponding author (IN). Requests to access the datasets should be directed to Inga.Neumann@biologie.uniregensburg.de.

## Ethics Statement

The animal study was reviewed and approved by the Local Government of Oberpfalz and Unterfranken.

## Author Contributions

VO designed, performed, analyzed the experiments, and prepared the first draft of the manuscript and of all figures. TJ supervised the project. TJ and IN substantially revised the manuscript and conceived the project. All authors contributed to the article and approved the submitted version.

## Conflict of Interest

The authors declare that the research was conducted in the absence of any commercial or financial relationships that could be construed as a potential conflict of interest.

## Publisher’s Note

All claims expressed in this article are solely those of the authors and do not necessarily represent those of their affiliated organizations, or those of the publisher, the editors and the reviewers. Any product that may be evaluated in this article, or claim that may be made by its manufacturer, is not guaranteed or endorsed by the publisher.
